# Supplementary material for: Dominant α-tubulin mutations rescue tauopathy neurodegenerative phenotypes in C. elegans
Source: bioRxiv. 2026 Mar 20:2026.03.18.712642. Preprint. [Version 1] doi: 10.64898/2026.03.18.712642 (PMC13015788; doi:10.64898/2026.03.18.712642)
Supplement: 1 [file NIHPP2026.03.18.712642v1-supplement-1.pdf]

bioRxiv preprint doi: https://doi.org/10.64898/2026.03.18.712642; this version posted March 20, 2026. The copyright holder for this preprint (which was not certified by peer review) is the author/funder, who has granted bioRxiv a license to display the preprint in perpetuity. It is made available under aCC-BY 4.0 International license.

990 **SUPPLEMENTARY MATERIAL**

991 **Supplementary Table 1. Mutant tubulin Alleles identified in forward genetic screening**

Gene name	Strain name	AA change	Codon change
mec-12	CK731	D431N	GAT-->AAT
tba-1	Ck1753, CK2050	E437K	GAG-->AAG
	CK2051	A429V	GCT-->GTT
	CK2192	E436K	GAA-->AAA
	CK4295	R425H	CGT-->CAT
tba-2	CK1458, CK1841	D429A	GAC-->GCC
	Ck1527, CK732	D429N	GAC-->AAC
	CK1782	V433D	GTC-->GAC
	CK2194	A424V	GCT-->GTT

Supplementary Table 2. Strain List

Strain abbreviations	Strain name	Strain genotype	Markers	Features
**Tau(V337M)**	CK10	*blks10[Paex-3::Tau V337M+Pmyo-2::GFP]*	Pmyo-2::GFP	Mutant tau, high expression
**WT-TauH**	CK144	*bkls144[Paex-3::Tau WT 4R1N+Pmyo-2::GFP]*	Pmyo-2::GFP	Wildtype tau, high expression
**WT-TauL**	CK1441	*bkls1441[Paex-3::Tau WT (4R1N)+Pmyo-2::dsRED]*	Pmyo-2::dsRED	Wildtype tau, low expression
**WT-TauM**	CK1443	*bkls1443[Paex-3::Tau WT (4R1N)+Pmyo-2::dsRED]*	Pmyo-2::dsRED	Wildtype tau, moderate expression
***tba-2* (D429A)*;* WT-TauH**	CK1458	*tba-2(bk1458); bkIs144[Paex-3::Tau WT (4R1N)+Pmyo-2::GFP]*	Pmyo-2::GFP	*tba-2* tau suppressor mutant
***tba-2* (D429N)*;* WT-TauH**	CK1527	*tba-2(bk1527); bkIs144 [Paex-3::Tau WT (4R1N)+Pmyo-2::GFP]*	Pmyo-2::GFP	*tba-2* tau suppressor mutant
***tba-1* (E437K)*;* WT-TauM**	CK1753	*tba-1(bk1753); bkIs1443[Paex-3::Tau WT (4R1N)+Pmyo-2::dsRED]*	Pmyo-2::dsRED	*tba-1* tau suppressor mutant
***tba-2* (V433D)*;* WT-TauM**	CK1782	*tba-2(bk1782); bkIs1443[Paex-3::Tau WT (4R1N)+Pmyo-2::dsRED]*	Pmyo-2::dsRED	*tba-2* (V433D) suppressor, Moderate wildtype human tau expression
**TDP-43**	CK1943	*bkIS1943[Psnb-1::hTDP-43 (WT)::K4aptazyme:unc-54 3'UTR+Pmyo-3::mCherry]*	Pmyo-3::mCherry	Expression of human TDP-43
***tba-2* (Tg-A)**	CK2111	*bkIs2111[Psnb-1::tba-2 (D429N)+Pmyo-3::mCherry]*	Pmyo-3::mCherry	Overexpression of neuronal *tba-2 (D429N)*
***tba-2* (Tg-B)**	CK2113	*bkIs2113[Psnb-1::tba-2 (D429N)+Pmyo-3::mCherry]*	Pmyo-3::mCherry	Overexpression of neuronal *tba-2 (D429N)*
***tba-2* (D429N)*;* Tau(V337M)**	CK2156	*tba-2(bk1527); bkIs10[Paex-3:: human Tau (4R1N) V337M+Pmyo-2::GFP]*	Pmyo-2::GFP	*tba-2* (D429N) with mutant human tau (V337M)
***tba-2* (Tg-A);WT-TauH**	CK2307	*bkIs2111[Psnb-1::tba-2 (D429N)+Pmyo-3::mCherry]; bkIs1443[Paex-3::tau WT (4R1N)+Pmyo-2::dsRED]*	Pmyo-3::mCherry; Pmyo-2::GFP	Overexpression neuronal expression of *tba-2* (D429N) and Moderate Expression of WT-Tau
***tba-2* (Tg-B);WT-TauH**	CK2308	*bkIs2113[Psnb-1::tba-2 (D429N)+Pmyo-3::mCherry]; bkIs1443[Paex-3::tau WT (4R1N)+Pmyo-2::dsRED]*	Pmyo-3::mCherry; Pmyo-2::GFP	Overexpression neuronal expression of *tba-2* (D429N) and Moderate Expression of WT-Tau
***tba-2* (D429N)*;* WT-TauL;TDP-43**	CK2484	*tba-2(bk1527); bkIs1441[Paex-3::Tau WT (4R1N)+Pmyo-2::dsRED]; bkIS1943[Psnb-1::hTDP-43 (WT)::K4aptazyme:unc-54 3'UTR+Pmyo-3::mCherry]*	Pmyo-2::dsRED; Pmyo-3::mCherry	Co-expression of human tau (low), human TDP-43 with mutant *tba-2*

***tba-7* (Q230stop)*;* WT-TauH**	CK2557	*tba-7(gk787939); bkIs144[Paex-3::Tau WT (4R1N)+Pmyo-2::GFP]*	Pmyo-2::GFP	Tubulin truncation mutant with tau expression
***tba-2* (D429N)**	CK2578	*tba-2(bk1527)*	unmarked	*tba-2(D429N)* suppressor mutant without tau
***mec-12* (D431N)*;* WT-TauH**	CK731	*mec-12(bk731); bkIs144[Paex-3::Tau WT (4R1N)+Pmyo-2::GFP]*	Pmyo-2::GFP	*mec-12* tau suppressor mutant
**Aβ**	CL2355	*smg-1(cc546);dvIs50 [pCL45 (snb-1::Abeta 1-42::3' UTR(long)+Pmtl-2::GFP]*	Pmtl-2::GFP	Expression of human Aβ1-42
**CZ1200**	CZ1200	*juIs76 [unc-25p::GFP+lin-15(+)]*	Unc-25::GFP	GFP labeled GABAergic ventral cord neurons, crossed with CK1527 and CK731 to visualize GABAergic ventral cord neurons
**WT Non-Tg**	N2	wildtype	NA	WT control
***tba-7* (Q230stop)**	VC40740	*tba-7(gk787939)*	unmarked	*tba-7* premature stop Q230* Tubulin truncation mutant
***mec-12* (D431N)**	CK4317	*mec-12(bk731)*	unmarked	*mec-12* (D431N) tau suppressor mutant without tau
***tba-2* (D429A)**	CK4318	*tba-2(bk1458)*	unmarked	*tba-2* (D429A) suppressor mutant without tau
***tba-1* (E437K)**	CK4319	*tba-1(bk1753)*	unmarked	*tba-1* (E437K) suppressor mutant without tau
***tba-2* (V433D)**	CK4320	*tba-2(bk1782)*	unmarked	*tba-2* (V433D) suppressor mutant without tau
***tba-2* (D429N);TDP-43**	CK4321	*tba-2(bk1527); bkIs1943[Psnb-1::hTDP-43 (WT)::K4aptazyme:unc-54 3'UTR+Pmyo-3::mCherry]*	Pmyo-3::mCherry	Expression of human TDP-43 with *tba-2* (D429N)
**WT-TauL;TDP-43**	NLS19	*bkIs1441[Paex-3::Tau WT (4R1N)+Pmyo-2::dsRED]; bkIS1943[Psnb-1::hTDP-43 (WT)::K4aptazyme:unc-54 3'UTR+Pmyo-3::mCherry]*	Pmyo-2::dsRED; Pmyo-3::mCherry;	Low expression of human Tau, and expression of human TDP-43
**WT-TauH;Aβ**	CK4323	*smg-1(cc546); bkls144[Paex-3::Tau WT 4R1N+Pmyo-2::GFP]; dvIs50 [pCL45 (snb-1::Abeta 1-42::3' UTR(long) + Pmtl-2::GFP]*	Pmtl-2::GFP, Pmyo-2::GFP	Human Tau and human Aβ1-42 expression
***tba-2* (D429N);Aβ**	CK4324	*tba-2(bk1527); smg-1(cc546);dvIs50 [pCL45 (snb-1::Abeta 1-42::3' UTR(long)+Pmtl-2::GFP]*	Pmtl-2::GFP	*tba-2* (D429N), and human Aβ1-42 expression
***tba-2* (D429N);WT-TauH;Aβ**	CK4325	*tba-2(bk1527); smg-1(cc546); bkls144[Paex-3::Tau WT 4R1N+Pmyo-2::GFP]; dvIs50 [pCL45 (snb-1::Abeta 1-42::3' UTR(long)+Pmtl-2::GFP]*	Pmtl-2::GFP, Pmyo-2::GFP	Human Tau expression, human TDP-43 expression, *tba-2* (D429N)

bioRxiv preprint doi: https://doi.org/10.64898/2026.03.18.712642; this version posted March 20, 2026. The copyright holder for this preprint (which was not certified by peer review) is the author/funder, who has granted bioRxiv a license to display the preprint in perpetuity. It is made available under aCC-BY 4.0 International license.

Supplementary Table 3. List of antibodies and dilutions used

Antibody	Antigen	Host Species	Source	Ref #	Application	Dilution
Thermo SP70	Pan-tau	Rabbit	ThermoFisher	MA5-16404	Western Blot<br><br>PeggySue	Total Tau: 1:2500<br>RAB/RIPA: 1:2000<br>FA tau 1:1000<br><br>1:2000
DAKO	Pan-tau	Rabbit	DAKO/Agilent	A0024(01-2)	Western blot<br><br>PeggySue	Total tau: 1:500,000<br>RAB/RIPA: 1:400,000<br>(Unsuitable for FA tau)<br><br>1:10,000
SP70 pAb	Pan-tau	Rabbit	Rockland	200-c)1-b33	Western Blot	Total Tau: 1:2500
PHF1	Phospho-tau p-ser396/Ser404	Mouse	Peter Davies	NA	Western Blot	1:1000
CP13	Phospho-tau	Mouse	Peter Davies	NA	Western Blot	1:1000
AT180	Phospho-tau	Mouse	ThermoFisher	MN040	Western Blot	1:1000
β-Tubulin	E7 mAb	Mouse	Developmental Studies Hybridoma Bank (Iowa City IA, USA)	NA	Western Blot	1:5000
2° Ab mouse	Horseradish Peroxidase α-Ms IgG (H + L)	Goat	Jackson Immunoresearch (West Grove, PA, USA)	115-035-146	Western Blot<br><br>PeggySue	1:5000<br><br>1:100
2° Ab Rabbit	Horseradish Peroxidase α-Rb IgG (H + L)	Mouse	Jackson Immunoresearch (West Grove, PA, USA)	211-032-171	Western Blot<br><br>PeggySue	1:5000<br><br>1:100

bioRxiv preprint doi: https://doi.org/10.64898/2026.03.18.712642; this version posted March 20, 2026. The copyright holder for this preprint (which was not certified by peer review) is the author/funder, who has granted bioRxiv a license to display the preprint in perpetuity. It is made available under aCC-BY 4.0 International license.

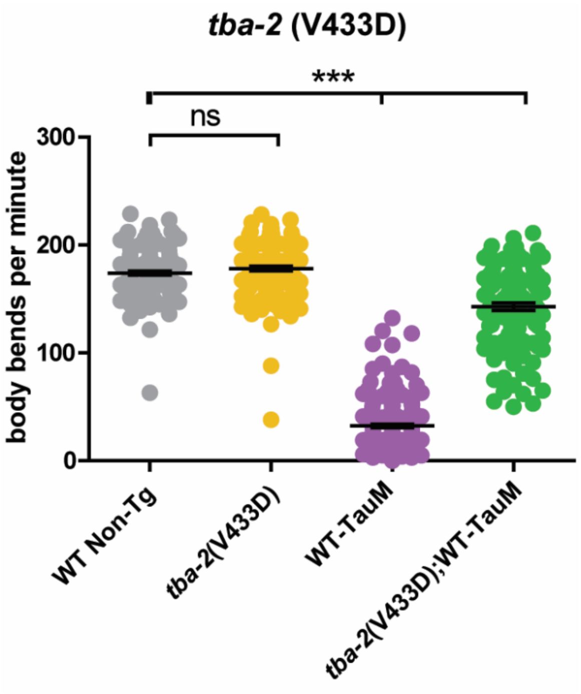

Supplementary Figure 1. *tba-2*(V433D) shows moderate levels of suppression in a tau-transgenic strain expressing medium levels of wildtype human 4R1N tau (CK1443). (***p<0.0001, Kruskal-Wallis ANOVA with Dunn's comparison, n≥114, error bars represent SEM).

bioRxiv preprint doi: https://doi.org/10.64898/2026.03.18.712642; this version posted March 20, 2026. The copyright holder for this preprint (which was not certified by peer review) is the author/funder, who has granted bioRxiv a license to display the preprint in perpetuity. It is made available under aCC-BY 4.0 International license.

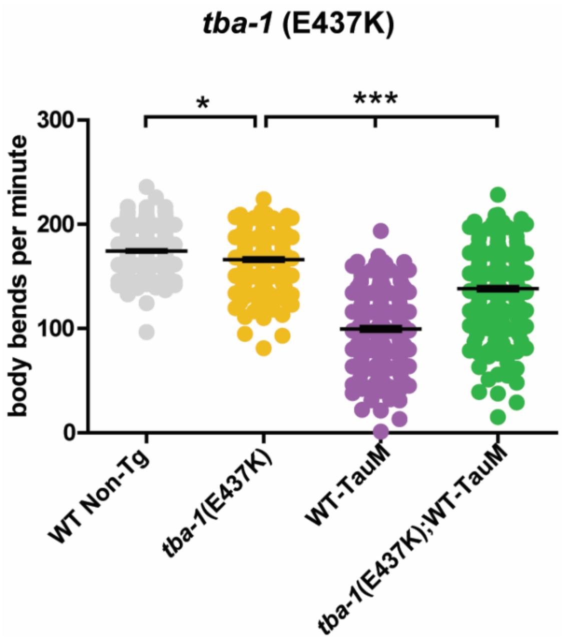

Supplementary Figure 2. Mutant *tba-1* moderately suppresses tau-induced motility deficits. *tba-1*(E437K) shows moderate levels of suppression in a tau-transgenic strain expressing medium levels of wildtype human 4R1N tau (CK1443). (***p<0.0001, Kruskal-Wallis ANOVA with Dunn’s comparison, n≥230, error bars represent SEM).

bioRxiv preprint doi: https://doi.org/10.64898/2026.03.18.712642; this version posted March 20, 2026. The copyright holder for this preprint (which was not certified by peer review) is the author/funder, who has granted bioRxiv a license to display the preprint in perpetuity. It is made available under aCC-BY 4.0 International license.

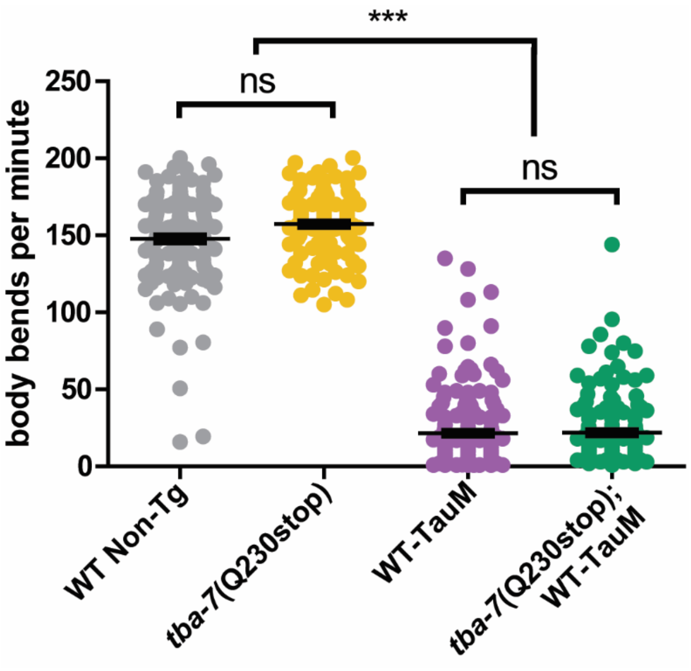

Supplementary Figure 3 Truncated tubulin does not rescue tau-induced phenotypes. The tubulin truncation of *tba-7* at Q230 does not rescue tau-induced motility deficits in worms expressing wildtype human tau at moderate expression levels (CK1443). Kruskal-Wallis ANOVA with Dunn's post-hoc test, ****p<0.0001*, n ≥ 146, error bars reflect SEM.

bioRxiv preprint doi: https://doi.org/10.64898/2026.03.18.712642; this version posted March 20, 2026. The copyright holder for this preprint (which was not certified by peer review) is the author/funder, who has granted bioRxiv a license to display the preprint in perpetuity. It is made available under aCC-BY 4.0 International license.

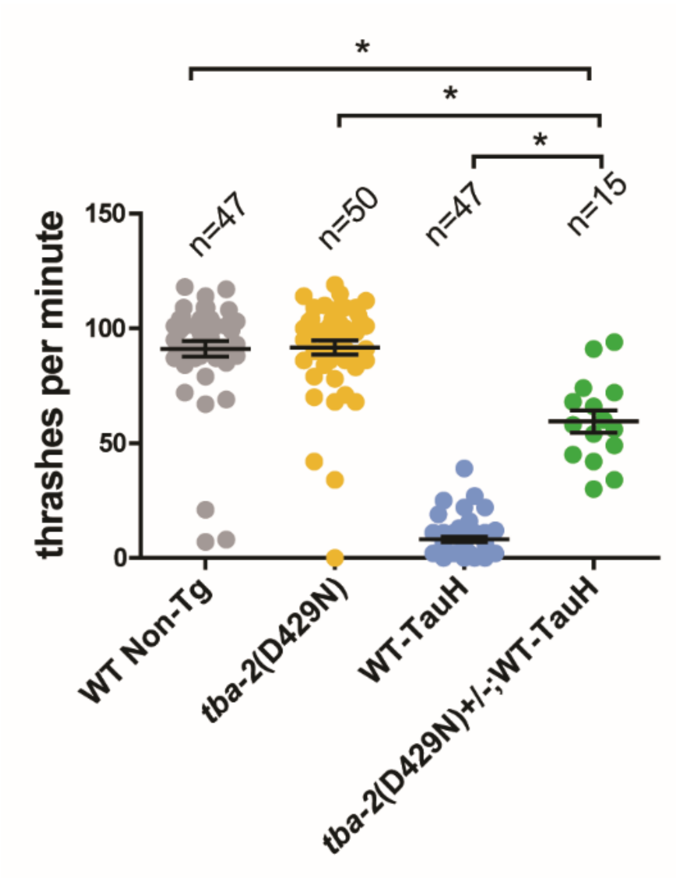

Supplementary Figure 4. Tubulin mutations are strong semi-dominant suppressors of tau toxicity. *C. elegans* homozygous for tau, but heterozygous for the *tba-2(D429N)* were tested for homozygous tau suppression in a swimming assay. Population is low due to post-swim confirmation of heterozygosity. n ≥ 15, Kruskall-Wallis ANOVA with Dunn's post-hoc test, *$p<0.05$* error bars reflect SEM.

bioRxiv preprint doi: https://doi.org/10.64898/2026.03.18.712642; this version posted March 20, 2026. The copyright holder for this preprint (which was not certified by peer review) is the author/funder, who has granted bioRxiv a license to display the preprint in perpetuity. It is made available under aCC-BY 4.0 International license.

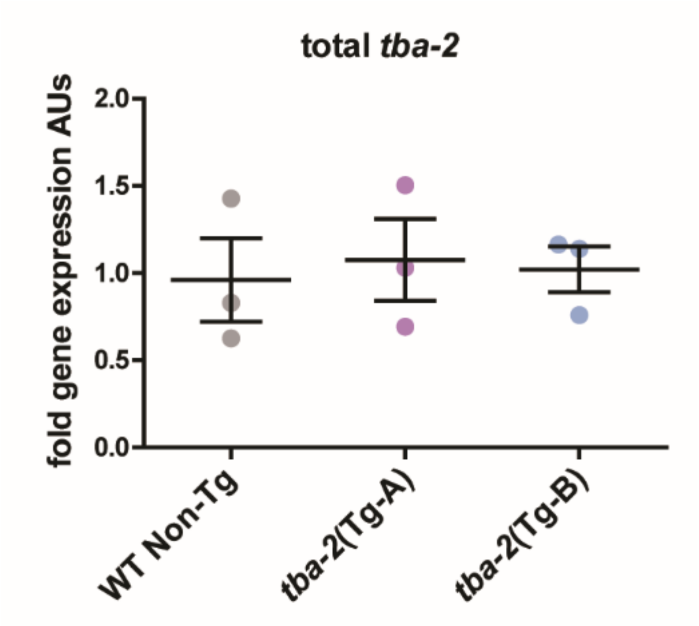

Supplementary Figure 5. Pan-tissue control for total *tba-2* mRNA in qPCR experiments. Primers amplified endogenous *tba-2* expressed across all worm tissues which remains unchanged since overexpression of mutant tubulin is specific to neurons that make up only a small portion of total *C. elegans* cells.

bioRxiv preprint doi: https://doi.org/10.64898/2026.03.18.712642; this version posted March 20, 2026. The copyright holder for this preprint (which was not certified by peer review) is the author/funder, who has granted bioRxiv a license to display the preprint in perpetuity. It is made available under aCC-BY 4.0 International license.

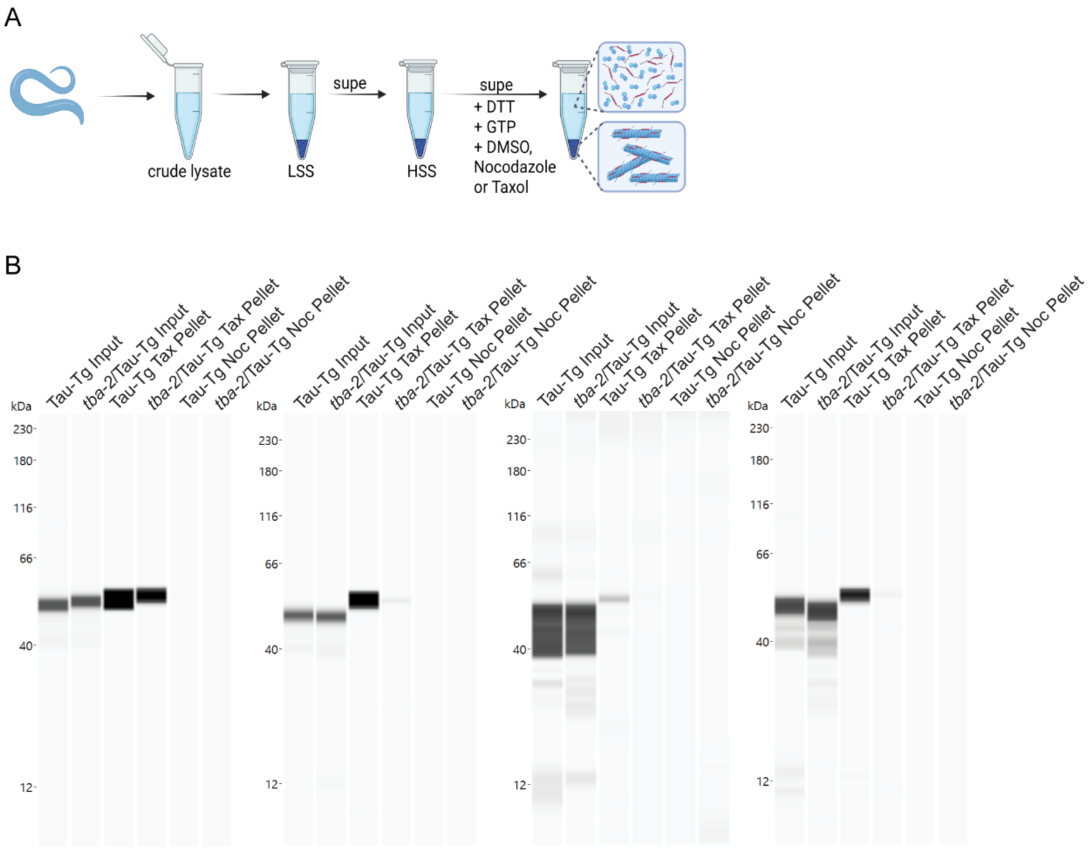

Supplementary Figure 6. A. Workflow schematic for sedimentation assay [Created in BioRender. Wheeler, J. (2025) https://BioRender.com/fqyruiy] Biological replicates of capillary westerns data showing input tubulin, and sedimented microtubule mass after incubation with taxol or nocodazole.

bioRxiv preprint doi: https://doi.org/10.64898/2026.03.18.712642; this version posted March 20, 2026. The copyright holder for this preprint (which was not certified by peer review) is the author/funder, who has granted bioRxiv a license to display the preprint in perpetuity. It is made available under aCC-BY 4.0 International license.

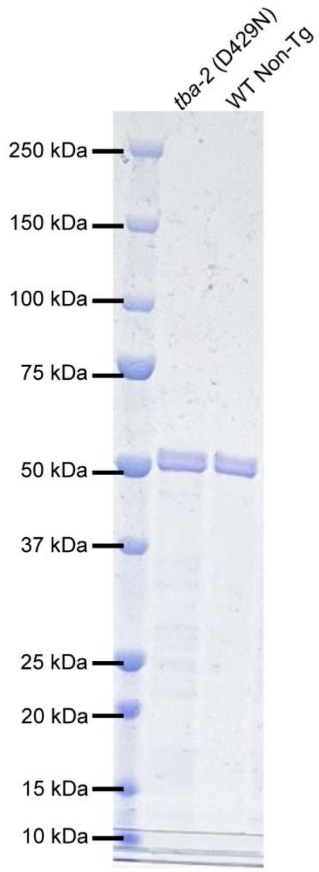

Supplementary Figure 7. Representative Coomassie stained acrylamide gel showing approximately 0.2mg/ml tubulin isolated from *tba-2* (D429N) and WT Non-Tg animals.
